# Myocardial Blood Flow and Metabolic Rate of Oxygen Measurement in the Right and Left Ventricles at Rest and During Exercise Using ^15^O-Labeled Compounds and PET

**DOI:** 10.3389/fphys.2019.00741

**Published:** 2019-06-19

**Authors:** Nobuyuki Kudomi, Kari K. Kalliokoski, Vesa J. Oikonen, Chunlei Han, Jukka Kemppainen, Hannu T. Sipilä, Juhani Knuuti, Ilkka H. A. Heinonen

**Affiliations:** ^1^Turku PET Centre, University of Turku, Turku, Finland; ^2^Department of Medical Physics, Faculty of Medicine, Kagawa University, Kagawa, Japan; ^3^Department of Clinical Physiology and Nuclear Medicine, University of Turku and Turku University Hospital, Turku, Finland; ^4^Rydberg Laboratory of Applied Sciences, University of Halmstad, Halmstad, Sweden

**Keywords:** positron emission tomography, myocardial blood flow, myocardial oxygen metabolism, right ventricle, left ventricle

## Abstract

**Aims:** Simultaneous measurement of right (RV) and left ventricle (LV) myocardial blood flow (MBF), oxygen extraction fraction (OEF), and oxygen consumption (MVO_2_) non-invasively in humans would provide new possibilities to understand cardiac physiology and different patho-physiological states.

**Methods:** We developed and tested an optimized novel method to measure MBF, OEF, and MVO_2_ simultaneously both in the RV and LV free wall (FW) using positron emission tomography in healthy young men at rest and during supine bicycle exercise.

**Results:** Resting MBF was not significantly different between the three myocardial regions. Exercise increased MBF in the LVFW and septum, but MBF was lower in the RV compared to septum and LVFW during exercise. Resting OEF was similar between the three different myocardial regions (~70%) and increased in response to exercise similarly in all regions. MVO_2_ increased approximately two to three times from rest to exercise in all myocardial regions, but was significantly lower in the RV during exercise as compared to septum LVFW.

**Conclusion:** MBF, OEF, and MVO_2_ can be assessed simultaneously in the RV and LV myocardia at rest and during exercise. Although there are no major differences in the MBF and OEF between LV and RV myocardial regions in the resting myocardium, MVO_2_ per gram of myocardium appears to be lower the RV in the exercising healthy human heart due to lower mean blood flow. The presented method may provide valuable insights for the assessment of MBF, OEF and MVO_2_ in hearts in different pathophysiological states.

## Introduction

Myocardial blood flow (MBF), oxygen extraction fraction (OEF) and metabolic rate of oxygen (MVO_2_) may be non-invasively assessed using ^15^O-water (${\text{H}}_{2}^{15}$O) and ^15^O-oxygen (^15^O_2_) with positron emission tomography (PET) (Iida et al., 1988, 1991, 1992, 1996; Yamamoto et al., 1996; Hermansen et al., 1998). The technique used for MBF and MVO_2_ calculation utilizes the single tissue compartment model, and its validity has been confirmed in several studies (Huang et al., 1985; Iida et al., 1988, 1991, 1992, 1996, 2000a; Yamamoto et al., 1996; Hermansen et al., 1998; Choi et al., 1999; Watabe et al., 2005). A unique feature of this technique is that the model incorporates the concept of the perfusable tissue fraction (PTF), which allows correction in MBF and thus also in MVO_2_ computation for partial volume effect (PVE) due to cardiac wall motion and the thin ventricular wall relative to the intrinsic spatial resolution of a PET scanner used (Iida et al., 1988, 1991).

For the left ventricle (LV) myocardium, validated techniques have been published to measure MBF and PTF applying dynamic ${\text{H}}_{2}^{15}$O PET scan (Watabe et al., 2005). The MBF measurements of the right ventricle (RV) are challenging with all imaging techniques due to complex shape of the chamber, thin wall, and its rapid motion. A technique to obtain non-invasive and direct measurement of quantitative MBF, PTF, and subsequent OEF and MVO_2_ in the RV together with the LV would be of importance, as it could for instance open up new insights for the evaluation of initiation, progression, and effectiveness of the treatments of various pathological states that not only affect LV but also often the RV (Voelkel et al., 2006). RV coronary perfusion and oxygen consumption are the major determinants of its function (Voelkel et al., 2006), and to the best of our knowledge MBF, OEF, and MVO_2_ in the human RV have been measured only in pulmonary hypertensive patients (Bokhari et al., 2011; Wong et al., 2011a,b,c), but never in healthy human subjects and also never simultaneously compared against to those of LV. Furthermore, in previous studies analyses to estimate RV blood curve were performed without corrections for PVE and spillover from surrounding myocardium to cavity (Wong et al., 2011a,b,c), which would be critical sources of possible errors.

Along these lines, the present study aimed to measure MBF, OEF and MVO_2_ in healthy human subjects in LV and RV myocardia simultaneously at rest and during exercise by developing a novel analysis method, which allows simultaneous computation of MBF and PTF in RV as well as LV walls from dynamic ${\text{H}}_{2}^{15}$O PET scan data and also to measure OEF and MVO_2_ from steady-state ^15^O_2_ scan data. Validity of the present method was tested by (1) comparing quantitative MBF values with those by the previous method which applied RV region of interest (ROI) based time activity curve (TAC) instead of RV blood TAC (Hermansen et al., 1998), (2) comparing MBF value in LV free wall (FW) myocardium at rest with during supine cycling exercise conditions, and (3) comparing PTF obtained from ${\text{H}}_{2}^{15}$O scan data with myocardial extravascular density from transmission scan data (Iida et al., 2000b). The validity was additionally tested by (4) comparing LV and RV blood volume values obtained from the ${\text{H}}_{2}^{15}$O and from C^15^O scan data.

## Materials and Methods

### RV Blood TAC Formula

To obtain the RV blood TAC, two factors of PVE in RV region of interest (ROI) TAC and spill-over effect from surrounding myocardial wall and adjacent organs are required to be corrected. Similar to the formulation for LV blood TAC, *C*_A, L_(*t*) (Iida et al., 1988), the formula for venous blood TAC in right ventricle, *C*_V, R_(*t*), was obtained as Equation (5) in the section Appendix A.

### Subjects

Healthy young men (*n* = 15, age 30 ± 5 years, height 179 ± 5 cm, weight 75 ± 7 kg, and maximal oxygen consumption 40 ± 5 mL/kg/min) were studied. The subjects were healthy as determined by health questionnaire and physical examination by a doctor in addition to pre-ECG and cardiac echocardiography evaluation. The subjects were not under any medication and were normotensive non-smokers with no history of hypercholesterolemia and no family history of coronary disease. The purpose, nature and potential risks were verbally explained to the subjects before they gave their written informed consent to participate. The study was performed according to the Declaration of Helsinki and was approved by the Ethical Committee of the Hospital District of South-Western Finland.

### PET Experiments

PET acquisition was carried out in 2D mode. The PET methods and protocols are explained in detail in our previous study (Heinonen et al., 2014). Briefly, after the transmission scan, scans were undertaken with ${\text{H}}_{2}^{15}$O bolus injection, C^15^O inhalation, and ^15^O_2_ continuous inhalation at the resting condition. Then, during exercise with supine cycling (100 Watts), scans with those were repeated. Due to problems in ^15^O_2_ or C^15^O tracer production, three subjects were missing the ^15^O_2_ or C^15^O scans and the present data are reported in 12 subjects.

### Data Processing

Images were reconstructed by the OSEM method using a Hann filter with a cut-off frequency of 4.6 mm. All data sets for same subjects were resliced using the same set of parameters. ROIs were drawn on LV and RV regions and LV and RV ROI TACs were obtained. ROIs for the left ventricle free wall (LVFW), septum and RV wall were also drawn and their TACs were extracted. Then the LV blood TAC, *C*_A, L_(*t*), was estimated using the previous method [Equation (3) in the section Appendix A] (Iida et al., 1988). The RV blood curve, *C*_V, R_(*t*), was estimated by using the present developed formulae [Equation (5) in the section Appendix A].

To generate MBF, PTF, and *V*_B, L_ and *V*_B, R_ images with Equation (7) in the section Appendix B, we applied obtained *C*_A, L_(*t*) and *C*_V, R_(*t*): *N*-method, thus those allow correction for spillover into the LV and RV myocardial walls (Hermansen et al., 1998). Details of the computation method are described in the section Appendix B. Also, the RV ROI curve instead of *C*_V, R_(*t*) was used for computing the those images: *H*-method (Hermansen et al., 1998). The blood volume image (*V*_B_) was also computed using the C^15^O scan data. The extravascular tissue density (*D*_ev_) [Equation (8) in the section Appendix B] (Iida et al., 1992), and perfusable tissue index (PTI) (Iida et al., 1992; Silva et al., 1992; Herrero et al., 1995), are computed using the *V*_B_ and reconstructed transmission image data. Using the obtained MBF and PTF, then, OEF and MVO_2_ in the LVFW, septal and RV wall regions were computed by applying the previously developed formulae (Iida et al., 1996; Yamamoto et al., 1996; Lubberink et al., 2010; Heinonen et al., 2014).

### Statistical Analysis

Data are shown as mean ± SD across subjects. The Student's paired *t*-test was used for the comparison of changes in basic hemodynamical variables from rest to exercise, and comparison between *N*- and *H*-methods. Two-way ANOVA for repeated measures was performed to assess the effects of exercise and regional differences in myocardial circulatory variables.

## Results

Basic hemodynamic variables at rest and during exercise are shown in Table 1. Exercise increased heart rate, systolic blood pressure, diastolic blood pressure, and thus as a result also the mean arterial pressure and rate pressure product.

**Table 1:** Hemodynamical variables at rest and during exercise obtained simultaneously with PET scanning.

	**Rest**	**Exercise**
Heart rate (bpm)	68 ± 8	134 ± 16^***^
BPs (mmHg)	123 ± 9	146 ± 13^***^
BPd (mmHg)	72 ± 6	80 ± 12^**^
MAP (mmHg)	89 ± 7	102 ± 6^***^

*Bpm, beats per minute; BPs, systolic blood pressure; BPd, diastolic blood pressure; MAP, mean arterial pressure*.

** p < 0.01 and

*** *p < 0.0001 compared to rest*.

**Figure 1:**
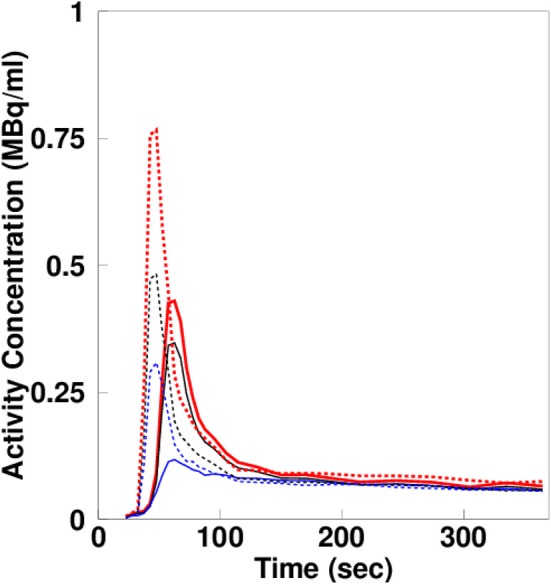
Estimated left and right ventricle blood curves, extracted left and right ventricle curves, and left and right myocardial activity curves.

Figure 1 shows representative TACs from LV and RV chamber regions, LV and RV myocardial regions, and estimated *C*_A, L_(*t*) and *C*_V, R_(*t*), respectively. The TACs in the right regions appear earlier than those in the left, reflecting that ${\text{H}}_{2}^{15}$O was infused into vein and thus firstly passed through RV and then continue via pulmonary circulation into LV. The estimated RV blood curve, *C*_V, R_(*t*), is higher in scale and less dispersed than left one, *C*_A, L_(*t*).

**Figure 2:**
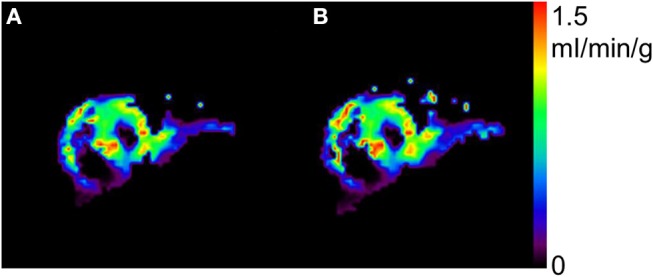
Representative view of myocardial blood flow by the present *N*- **(A)** and previous *H*-methods **(B)**.

**Figure 3:**
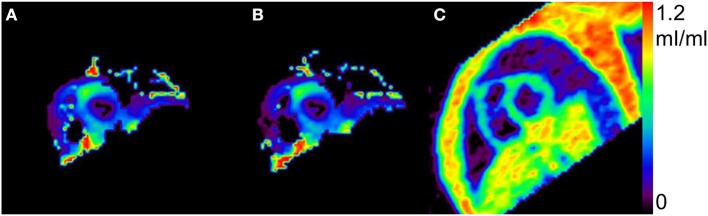
Representative view of perfusable tissue fraction by the present *N*- **(A)**, the previous *H*-methods **(B)**, and extravascular tissue density D_*ev*_ **(C)**.

**Figure 4:**
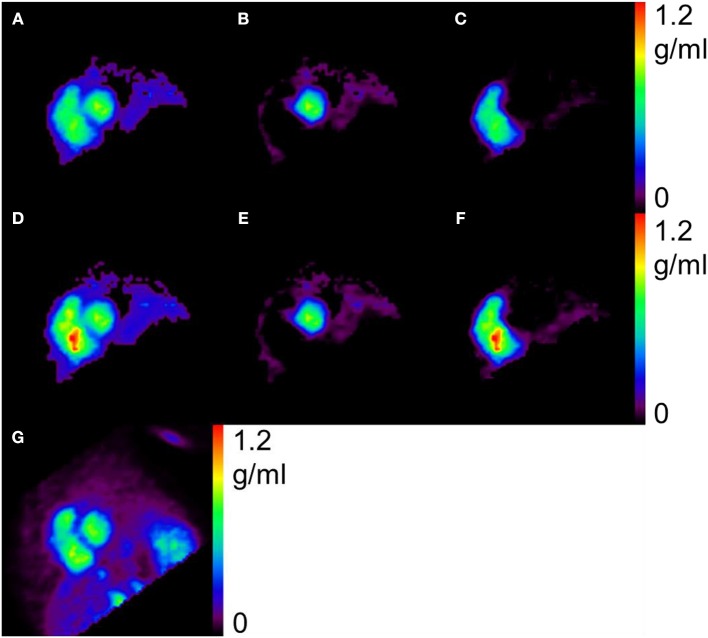
Representative view of blood volume, by the present *N*- **(A–C)**, the previous *H*-methods **(D–F)**, and C15O scan **(G)**. The left **(B,E)** and right **(C,F)** ventricles are clearly separated and the blood volume **(A)** as a sum of **(B)** and **(C)** is similar to that by the C15O scan **(G)**.

Figure 2 shows a representative view of MBF in one of the subjects at the resting condition by the *N*- and *H*-methods. The MBF in LVFW, septal and RV walls can be seen in the figure, and the shape and contrast are similar in the LV region between *N*- and *H*-methods. Figure 3 compares PTF images by the *N*- and *H*-methods, and *D*_ev_ image. The LVFW, septal and RV wall can be seen in the images, and in LVFW region, the shape and contrast are similar to those by the *H*-method, and in RV wall PTF seems lower in *H*-method. Also, shape in PTF is similar to the *D*_ev_ image. Blood volume images are shown in Figure 4. The left and right ventricle regions are clearly separated in *V*_B, L_ and *V*_B, R_ images. The *V*_B, L_+*V*_B, R_ image by the *N*-method is similar to the *V*_B_ image by the C^15^O scan method, however, that seems higher in RV region in the *H*-method.

**Figure 5:**
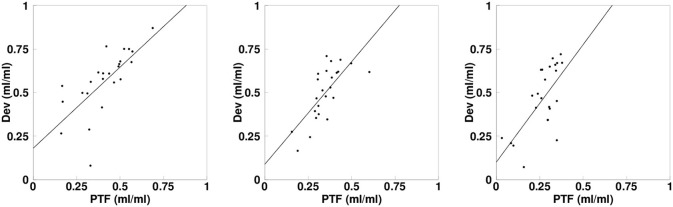
Linear regressions depicting the correlation between the perfusable tissue fraction (PTF) and the extravascular tissue density (*D*_ev_) values for LV (L), septum (S), and RV (R). The solid lines show the regressions.

Quantitative values of MBF, PTF, *D*_ev_, and PTI are summarized in Table 2, those of blood volumes are in Table 3, and OEF and MVO_2_ in Table 4. The MBF and PTF values based on the *N*- and *H*-methods were not significantly different in any of the myocardial regions. There were however significant differences between PTF and *D*_ev_ for all regions. Regression analysis showed however correlation between PTF and *D*_ev_ for all LVFW (*r* = 0.71, *P* < 0.001), septum (*r* = 0.72, *P* < 0.001) and RV wall (*r* = 0.67, *P* < 0.001) (Figure 5. The corresponding PTI values in resting condition were between 0.6–0.7. For the blood volume values, there were no significant differences between *N*-method and C^15^O scan method, either in LV and RV (Table 3).

**Table 2:** Parameters in myocardial region calculated using the *N*- and *H*-methods (*n* = 12).

	**Resting condition**			**Exercise condition**		
	**Left**	**Septum**	**Right**	**Left**	**Septum**	**Right**
MBF (N) (mL/min/g)	0.92 ± 0.24	0.91 ± 0.21	1.07 ± 0.25	2.58 ± 0.55^***^	2.49 ± 0.38^***^	1.57 ± 0.60^##^
MBF (H) (mL/min/g)	0.93 ± 0.25	0.92 ± 0.21	1.13 ± 0.26^#^	2.60 ± 0.52^***^	2.56 ± 0.40^***^	1.51 ± 0.56^##^
PTF (N) (mL/mL)	0.46 ± 0.07^†^	0.46 ± 0.08^†^	0.25 ± 0.08^###^, ^†^	0.38 ± 0.08^*^, ^†^	0.43 ± 0.11^*^, ^†^	0.19 ± 0.09^*^, ^*###*^, ^†^
PTF (H) (mL/mL)	0.45 ± 0.07^†^	0.43 ± 0.09^&&^,^†^	0.22 ± 0.08^&&^,^*###*^, ^†^	0.38 ± 0.08^**^, ^†^	0.41 ± 0.11^*^, ^†^	0.14 ± 0.09^*^, ^*####*^, ^†^
*D*_ev_ (mL/mL)	0.62 ± 0.07	0.64 ± 0.12	0.49 ± 0.18^##^	0.62 ± 0.09	0.64 ± 0.09	0.46 ± 0.10^##^
PTI (N)	0.68 ± 0.22	0.70 ± 0.44^&&&^	0.66 ± 0.19^&&^	0.60 ± 0.10^*^	0.66 ± 0.22^*^	0.40 ± 0.11^*^,^&&^
PTI (H)	0.66 ± 0.22	0.60 ± 0.18	0.55 ± 0.24	0.62 ± 0.12	0.66 ± 0.20^**^	0.27 ± 0.15^**^, ^*###*^

MBF, myocardial blood flow; PTF, Perfusable tissue fraction; PTI, Perfusable tissue index; D_ev_, extravascular tissue density; N and H, N-and H-methods (see methods for details).

*, *** : difference was significant (P < 0.05, P < 0.001, respectively) between resting and exercise conditions.

&&, &&& : difference was significant (P < 0.01 and P < 0.001, respectively) between the N- and H-methods.

† : difference was significant (P < 0.001) compared to D_ev_.

##, ### *: difference was significant (P < 0.01 and P < 0.001, respectively) compared to left and septal regions*.

**Table 3:** Blood volume values in ventricle region estimated from the present *N*- and *H*- methods, and the C^15^O scan method (*n* = 12).

	**Resting condition**		**Exercise condition**	
	**LV**	**RV**	**LV**	**RV**
${\text{H}}_{2}^{15}$O				
*V*_B, L_+*V*_B, R_ (mL/mL) (*N*)	0.90 ± 0.03	0.88 ± 0.02^&&&^	0.93 ± 0.06	0.94 ± 0.06^&&&^
*V*_B, L_+*V*_B, R_ (mL/mL) (*H*)	0.90 ± 0.03	1.11 ± 0.02^†^	0.95 ± 0.05	1.23 ± 0.10^†^
C^15^O				
V_B_ mL/mL	0.90 ± 0.05	0.89 ± 0.06	0.93 ± 0.11	0.94 ± 0.11

N and H: computed by N- and H-methods.

&&& *: difference was significant (P < 0.001) between the N- and H-methods*.

† *: difference was significant (P < 0.001) between ${\text{H}}_{2}^{15}$O and C^15^O scan methods*.

**Table 4:** Oxygen extraction fraction (OEF) and myocardial oxygen consumption (MVO_2_) in different myocardial regions calculated using the *N*- and *H*-methods (*n* = 12).

	**Resting condition**			**Exercise condition**		
	**Left**	**Septum**	**Right**	**Left**	**Septum**	**Right**
*N*-method						
OEF	0.70 ± 0.08	0.71 ± 0.18^&^	0.73 ± 0.09^&&^	0.84 ± 0.14^***^	0.84 ± 0.10^***^	0.95 ± 0.06^***^
MVO_2_ (mL/min/g)	0.14 ± 0.04	0.13 ± 0.04	0.16 ± 0.05	0.44 ± 0.09^***^	0.44 ± 0.10^***^	0.30 ± 0.12^***^, ^#^
*H*-method						
OEF	0.68 ± 0.08	0.76 ± 0.19	0.88 ± 0.23^##^	0.84 ± 0.12^***^	0.93 ± 0.14^***^,^&&&^	1.25 ± 0.17^***^, ^*##*^,^&&&^
MVO_2_ (mL/min/g)	0.14 ± 0.04	0.15 ± 0.04	0.21 ± 0.08	0.46 ± 0.11^***^	0.49 ± 0.11^***^,^&&&^	0.77 ± 0.77^**^,^&^

**, *** : difference was significant (P < 0.01, P < 0.001, respectively) between resting and exercise conditions.

#, ## : difference was significant (P < 0.05, P < 0.01, respectively) compared to left and septal regions.

&, &&& *: difference was significant (P < 0.05, P < 0.001, respectively) between the N- and H-methods*.

The MBF was similar in all myocardial regions at rest based on the newly developed *N*-method. Exercise increased MBF significantly in the LVFW and septum, but MBF was significantly lower in the RV as compared to the LVFW and the septum (Table 2). Resting OEF was similar in all regions (Table 4) and OEF increased in response to exercise in all regions being the highest in the RV wall (Table 4). MVO_2_ increased two to three times from rest to exercise, but MVO_2_ was significantly lower in the RV as compared to the two other myocardial regions during exercise (Table 4). Finally, myocardial vascular resistance decreased from rest to exercise in all ventricular regions (*P* = 0.01), but was always higher in the RV (127 ± 37 at rest and 109 ± 96 mmHg/mL/min/g during exercise) compared to the LVFW myocardium (101 ± 25 at rest and 61 ± 32 mmHg/mL/min/g during exercise, *P* = 0.010) and the septum (95 ± 19 at rest and 72 ± 39 mmHg/mL/min/g during exercise, *P* = 0.015), which did not differ from each other (*P* = 0.99).

## Discussion

In the present study, we developed and tested a method which allowed estimating RV blood TAC with taking into account PVE and spillover effect, to simultaneously generate quantitative MBF and PTF images for RV and LV myocardial regions using ${\text{H}}_{2}^{15}$O PET data. Subsequently, OEF and MVO_2_ were computed using ^15^O_2_ PET data applying the obtained MBF and PTF. The present result showed that the MBF values estimated were similar between the present *N*-method and the previously established *H*-method (Hermansen et al., 1998). The obtained PTF values by the *N*-method were also similar to those analyzed by the *H*-method (Hermansen et al., 1998). Comparison in PTF and *D*_ev_ by the regression analysis showed tight correlations for all LVFW, septal and RV myocardial regions. The obtained blood volume values were not significantly different neither in LV nor RV between by the present *N*-method and the CO scan method. These findings suggest that the MBF and MVO_2_ values for both RV and LV myocardial regions are feasible to quantify and assess by the present method, and as such, the method could provide valuable insights for several cardiovascular disease states that affect either both, or primarily the right ventricle (Mertens and Friedberg, 2010).

In the present study, the obtained MBF values by the *N*-method were similar to those analyzed by the *H*-method (Hermansen et al., 1998), and similar also to the previously obtained mean values ranging from 0.8 to 1.2 mL/min/g in resting condition (Huang et al., 1985; Iida et al., 1988, 1991, 1992, 1996; Silva et al., 1992; Hermansen et al., 1998; Lubberink et al., 2010). Those results indicate the validity of the present MBF computation technique. The obtained PTF values by the *N*-method in the LVFW were also similar to those analyzed by the *H*-method (Hermansen et al., 1998). For the septal and RV wall region, the PTF values were slighty lower in the *H*-method but especially as compared to *D*_ev_ (Table 2). The difference was likely due to the difference of height between in RV blood TAC and in RV ROI TAC as shown in Figure 1. RV blood TAC was estimated with taking into account PVE and spillover effect from RV ROI TAC and was thus higher than RV ROI TAC. When RV ROI TAC was applied for the computation, blood volume in the RV was overestimated, being >1.0 mL/mL and was significantly higher than that obtained by the CO scan method. Subsequently, the subtraction on RV blood TAC in RV wall region was excessive, and therefor PTF estimated was smaller. Then the estimated OEF was higher than one in the *H*-method, which is not physiological. The present N-method OEF and MVO_2_ values for the RV myocardium were computed along with the previously demonstrated method developed for LV with applying MBF and PTF (Iida et al., 1996; Yamamoto et al., 1996), implementing correction for the PVE and spillover effect due to cardiac and respiratory motion, and the thin ventricular wall relative to the intrinsic spatial resolution of a PET scanner used (Iida et al., 1988, 1991). Those facts suggest that when quantitatively imaging the MBF, OEF, and MVO_2_ in myocardial region, it would be critical to apply the PVE and spillover effect correction to obtain RV blood curve.

PTF values in the LVFW and septal walls obtained in the study were smaller than those in the previous studies (Iida et al., 1991, 1992, 1996; Hermansen et al., 1998) This could be due to different size of ROI drawn on those regions, namely we intentionally drawn larger size of ROI in LV region in this study for validating MBF computation method in thinner RV wall. When we drew smaller size of ROI in septum region, PTF values estimated were 0.74 ± 0.08 mL/mL which is similar to the previous studies (Iida et al., 1991, 1992, 1996; Hermansen et al., 1998). Comparison in PTF and *D*_ev_ by the regression analysis however showed tight correlations for all LVFW, septal and RV wall: *r* = 0.87, *r* = 0.67, and *r* = 0.56, respectively. The PTF estimates density of myocardium and thus allows the partial volume correction. The *D*_ev_ is also a measure of density of myocardium [Equation (8) in the section Appendix B] (Iida et al., 1991). We found, however, a significant difference between PTF and *D*_ev_, and that the PTI values, which is ratio between them (Iida et al., 1991; Silva et al., 1992; Herrero et al., 1995), were not close to 1.0 but around 0.7. A previous simulation study demonstrated that the decrease of PTI from 1 to 0.75 is due to heterogeneity and shape of input function (Herrero et al., 1995). As mentioned, the size of ROI in the present study was larger and this could have enhanced the degree of heterogeneity. Those factors may affect the present PTI values to be smaller than 1. As a whole, however, the tight regional correlations and smaller value of PTI identical to the previous demonstration suggest quantitative accuracy of the present approach (Herrero et al., 1995).

For the quantitative computation of MBF in the RV myocardium, application of the RV blood TAC after spillover and PVE correction was crucial and the model for the RV blood TAC was obtained by remodeling the previous method for the LV (Iida et al., 1992). The validity of the RV blood TAC was tested by comparing generated V_B_ in addition to MBF, and PTF as above. Blood volume in both ventricular regions can be feasibly obtained by the C^15^O scan data (Watabe et al., 2005), and that was also estimated using the ${\text{H}}_{2}^{15}$O scan analysis by Harms et al. (2011). We also estimated the blood volume from the ${\text{H}}_{2}^{15}$O scan data in both LV and RV to test the validity of the present method for the RV blood TAC by comparing the volume between the two ${\text{H}}_{2}^{15}$O and C^15^O methods. The obtained blood volume values were not significantly different neither in LV nor RV. The LV and RV were clearly separated in the generated *V*_B, L_ and *V*_B, R_ images, suggesting reliability of the estimated RV blood TAC. A possible method to obtain the right blood TAC could be to use the RV ROI TAC (Hermansen et al., 1998), however, estimated blood volume in RV was significantly larger than that by the CO scan method, and furthermore the values were larger than 1 mL/mL (Table 3; Figure 3). Those also suggest that PVE correction is critical for the RV blood TAC, as far as a PET scanner with high spatial resolution is not used (Mertens and Friedberg, 2010).

### Physiological Considerations

It is very important to make it possible to measure RV myocardial parameters such as MBF, OEF and MVO_2_ non-invasively, because they are major determinant of RV function (Klima et al., 1999), which in turn is compromised in many patophysiological states (Voelkel et al., 2006). Based on the animal studies, RV MBF is typically 50–90% lower than that in the LV (Zong et al., 2005). In resting swine whose heart would be the closest to human heart, RV MBF is 70–90% of LV MBF (Duncker and Bache, 2008). However, our present PET MBF findings suggest that RV blood flow in humans is similar to that in the LV and septum. However, largely based on canine studies, but also one swine study (Schwartz et al., 1994), one feature of the RV myocardium compared to left one has been considered to be its markedly lower oxygen extraction fraction (40–50%) and subsequent metabolic rate (Klima et al., 1999; Zong et al., 2005; Duncker and Bache, 2008). In contrast, we observed that RV myocardial OEF tended to be higher than that in the LV, especially during exercise. Species difference is the most likely explanation for this difference. In fact, one previous human PET study has already reported that RV myocardial oxygen extraction appears to be much higher than observed in animals (Wong et al., 2011b), although that study was performed in pulmonary hypertensive patients and not in healthy human subjects. The current study reports for the first time that the normal myocardial oxygen extraction might be higher in healthy humans than previously found in animal studies.

It is well-established that RV MBF increases during exercise as a direct function of heart rate (Klima et al., 1999; Zong et al., 2005). In animal studies it has been found that RV MBF increases relatively more and can even exceed MBF in the LV at heavy exercise, as oxygen consumption increases relatively more secondary to the marked increase in pulmonary artery pressure close to maximal exercise intensity (Klima et al., 1999; Duncker and Bache, 2008). However, at lower exercise intensities, as also applied in the present study, pulmonary pressure remains close to resting values, while LV systolic pressure increases. Theoretically, this physiological background could lead to the situation that MBF, OEF as well as MVO_2_ increase relatively more in the LV than in septal and RV myocardial regions. Interestingly, in accordance with this idea we found in the present study that MVO_2_ computed by the newly developed N-method was significantly lower in the RV during exercise as compared to LVFW and septum, due to its lower blood flow. This is also in line with animal studies in which blunted RV myocardial blood flow response and large enhancement in oxygen extraction and consumption has been observed in response to exercise (Klima et al., 1999; Duncker and Bache, 2008). This has been at least in part explained by exaggerated α-adrenergic vasoconstrictor influence on the right ventricular vasculature (Klima et al., 1999). Interestingly, we also found in the present study that myocardial vascular resistance was higher in the RV compared to both LV and septal myocardia, which together with lower blood flow and resulting higher myocardial blood mean transit time is plausible mechanism to contribute to higher oxygen extraction in RV especially during exercise (Heinonen et al., 2014). Altogether, as the direct sampling of oxygen content in human RV myocardium is extremely difficult, the present technique would allow unique access to evaluate human RV blood flow and metabolic demand quantitatively, and has potential to provide mechanistic insights to numerous pathological states in terms of right ventricle (Iida et al., 2000b; Voelkel et al., 2006).

### Limitations

Due to the large amount of variables and methods comparisons and thus analyses burden to obtain even these results, only one researcher observed and analyzed the images. Thus, no intra- or inter-observer variation was documented in the present study, which should be addressed in the future studies. Further, no comprehensive echocardiographic analyses were also performed in the current study. Information of especially RV wall thicknesses could provide further insights especially in the pathophysiological states on the relation of structural aspects with these PET-derived circulatory and oxygen metabolism related variables.

## Summary and conclusions

In conclusion, this study presents a method to obtain MBF image, and OEF and MVO_2_ values simultaneously for both LV and RV myocardia using ${\text{H}}_{2}^{15}$O and ^15^O_2_ tracers and PET imaging. In addition we showed that the method developed was feasible in quantitative assessment of MBF, OEF and MVO_2_ in humans, at rest and physiologically challenging exercise condition. As such, the application of the method and model could provide valuable insights for the assessments of perfusion and various pathological states affecting RV (Mertens and Friedberg, 2010), such as in pulmonary hypertension, which possesses great challenges for the right ventricle.

## Ethics Statement

This study was carried out in accordance with recommendations of Ethics Committee of Hospital District of South-West Finland with written informed consent from all subjects. All subjects gave written informed consent in accordance with the Declaration of Helsinki. The protocol was approved By the Ethics Committee of the Hospital District of South-West Finland.

## Author Contributions

All authors listed have made a substantial, direct and intellectual contribution to the work, and approved it for publication.

### Conflict of Interest Statement

The authors declare that the research was conducted in the absence of any commercial or financial relationships that could be construed as a potential conflict of interest.

## Glossary

MBF: Myocardial blood flow
OEF: Oxygen extraction fraction
MVO_2_: Metabolic rate of oxygen
PET: Positron emission tomography
H_2_^15^O: ^15^O labeled water
^15^O_2_: ^15^O labeled oxygen
PTF: perfusable tissue fraction
PVE: partial volume effect
LV: Left ventricle
FW: Free wall
LVFW: Left ventricle free wall
ROI: region of interest
TAC: time activity curve
*N*-method: a method for RV-MBF computation developed in the present study
*H*-method: a method for RV-MBF computation developed in the previous study (Hermansen et al., 1998)
*C*_A,L_(*t*): LV blood TAC
*C*_V,R_(*t*): the venous blood TAC in right ventricle
*C*_T,R_(*t*): True myocardial tissue TAC
*D*_R_(*t*): ROI TAC in the right ventricle (Bq/mL)
*D*_m,R_(*t*): ROI TAC in RV myocardial region (Bq/mL)
*f*_R_: RV myocardial blood flow
β_R_: recovery coefficient in RV myocardial ROI (0.0 < β_R_ < 1.0)
γ_R_: spillover fraction in RV myocardial ROI (0.0 < γ_R_ < 1.0)
α_R_: PTF (g/mL)
*D*_ev_: Extravascular tissue density
*V*_B,L_: LV blood volume
*V*_B,R_: RV blood volume
*V*_B_: Blood volume
PTI: Perfusable tissue index
