# Supplementary material for: Myocardial Blood Flow and Metabolic Rate of Oxygen Measurement in the Right and Left Ventricles at Rest and During Exercise Using 15O-Labeled Compounds and PET
Source: Front Physiol. 2019 Jun 19;10:741. doi: 10.3389/fphys.2019.00741 (PMC6593089; doi:10.3389/fphys.2019.00741)
Supplement: Supplementary file 1 [file Data_Sheet_1.pdf]

Myocardial blood flow and metabolic rate of oxygen measurement in the right and left ventricles at rest and during exercise using ^{15}O-labeled compounds and PET

Authors

Nobuyuki Kudomi, Kari K. Kalliokoski, Vesa J. Oikonen, Chunlei Han, Jukka Kemppainen, Hannu T. Sipila, Juhani Knuuti, Ilkka Heinonen

Appendix

A. Formula for RV blood TAC

The tissue TAC of the cardiac region observed by PET ($C_T(t)$) after the administration of $H_2{}^{15}O$ can be expressed using MBF (f), PTF (α), and blood volume in LV ($V_{B,L}(t)$) and RV ($V_{B,R}$(t)), as follows (Iida H et al 1991, 1992):

$$C_T(t) = \alpha \cdot f \cdot C_A(t) \otimes e^{-\frac{f}{p} \cdot t} + V_{B,L} \cdot C_{A,L}(t) + V_{B,R} \cdot C_{V,R}(t) \qquad (1)$$

where $C_{A,L}(t)$ and $C_{V,R}(t)$ (Bq/mL) are the arterial and venous blood TACs in the LV and RV, respectively, p is the tissue/blood partition coefficient (assuming a constant of 0.91 mL/g (Iida et al 1992)), $\otimes$ denotes the convolution operation. $C_T(t)$ and $C_A(t)$ in Equation (1) are corrected for the physical decay of ^{15}O. The LV blood TAC can be used as an arterial input function for myocardial tissue in LV region, i.e., C_A(t)=$C_{A,L}(t)$, and that can be obtained by the previously established method, (Iida et al 1992) namely, from the following equations;

$$\begin{aligned} C_{T,L}(t) &= f_L \cdot C_{A,L}(t) \otimes e^{-\frac{f_L}{p} \cdot t} \\ D_L(t) &= \beta_L \cdot C_{A,L}(t) + \gamma_L \cdot \rho \cdot C_{T,L}(t) \\ D_{m,L}(t) &= \alpha_L \cdot f_L \cdot C_{A,L}(t) \otimes e^{-\frac{f_L}{p} \cdot t} + V_{B,L} \cdot C_{A,L}(t) \end{aligned} \qquad (2)$$

where, the first $C_{T,L}(t)$ represents the LV myocardial tissue TAC, the second $D_L(t)$ (Bq/mL) and the last $D_{m,L}(t)$ (Bq/mL) represent the TACs of region of interest (ROI) in the LV and the left myocardial regions, respectively, f_L is MBF in LV myocardium, β_L ($0.0 < \beta_L < 1.0$) and γ_L ($0.0 < \gamma_L < 1.0$) are the recovery coefficient and the spillover fraction of tissue radioactivity, respectively, in the LV ROI, where $\beta_L + \gamma_L = 1$, ρ is the myocardial tissue density (=1.04 g/mL) and α_L is the PTF (g/mL) of the left myocardial ROI. Solving the Equation (3), we obtain the LV blood TAC as:

$$\begin{aligned} C_{A,L}(t) &= \frac{1}{\beta_L}\cdot D_L(t) - \frac{1-\beta_L}{\beta_L^2}\rho\cdot f\cdot D_L(t)\otimes exp(-((\frac{1}{p}+\frac{1-\beta_L}{\beta_L})f)t) \\ D_{m,L}(t) &= (\frac{\alpha}{\beta_L}-\frac{1-\beta_L}{\beta_L^2}\rho\cdot V_{B,L})f\cdot D_L(t)\otimes exp(-((\frac{1}{p}+\frac{1-\beta_L}{\beta_L})f)t)+\frac{V_{B,L}}{\beta_L}\cdot D_L(t) \end{aligned} \quad (3)$$

Similar to equations (2) and (3), the RV blood TAC in right ventricle, $C_{V,R}(t)$, can be also estimated from the following equations, corresponding to RV;

$$\begin{aligned} C_{T,R}(t) &= f_R\cdot C_{A,L}(t)\otimes e^{-\frac{f_R}{p}\cdot t} \\ D_R(t) &= \beta_R\cdot C_{V,R}(t)+\gamma_R\cdot\rho\cdot C_{T,R}(t) \\ D_{m,R}(t) &= \alpha\cdot f_R\cdot C_{A,L}(t)\otimes e^{-\frac{f_R}{p}\cdot t}+V_{B,R}\cdot C_{V,R}(t) \end{aligned} \quad (4)$$

where $C_{T,R}(t)$ is the myocardial tissue TAC, $D_R(t)$ (Bq/mL) and $D_{m,R}(t)$ (Bq/mL) are the TACs of ROI which are selected in the RV and its wall, respectively, f_R is myocardial blood flow, β_R ($0.0 < \beta_R < 1.0$) and γ_R ($0.0 < \gamma_R < 1.0$) are the recovery coefficient and the spillover fraction of tissue radioactivity in RV ROI, where $\beta_R+\gamma_R = 1$, and α_R is PTF (g/mL) of the RV myocardial ROI. Solving the Equation (4), with noting that the input in the RV myocardial tissue is supplied via coronary arteries, we obtain the RV blood TAC, $C_{A,L}(t)$, as:

$$C_{V,R}(t)=\frac{1}{\beta_R}\cdot D_R(t)-\frac{1-\beta_R}{\beta_R{}^2}\rho\cdot f_R\cdot C_{A,L}(t)\otimes\exp(-\frac{1}{p}f_R\cdot t)$$
$$D_{m,R}(t)=(\alpha_R-\frac{1-\beta_R}{\beta_R{}^2}\rho\cdot V_{B,R})f_R\cdot C_{A,L}(t)\otimes\exp(-\frac{f_R}{p}t)+\frac{V_{B,R}}{\beta_R}\cdot D_R(t) \tag{5}$$

Using Equations (3) and (5), two of LV and RV blood TACs ($C_{A,L}(t)$ and $C_{V,R}(t)$) can be obtained.

B. Formulae for parametric imaging

We have applied a basis function method (BFM), which was introduced by Koeppe and Holden 1985 for cerebral blood flow imaging computation and has been applied for myocardial blood flow studies (Koeppe and Holden 1985; Watabe et al 2005; Boellaard et al 2005). This method allows dealing with non-linear term by choosing a discrete spectrum of parameter values for *f/p*. The corresponding basis function formed as;

$$F\,(f,t)\;=\;C_{A,L}\otimes e^{-\frac{f}{p}\cdot t} \tag{6}$$

In this study, the range of *f/p* was set to 0.0 < *f/p* < 5.0 /min, and 500 discrete basis functions were generated. Then Equation (1) can then be transformed for each basis function into a linear equation in $\Theta(=\alpha\cdot f)$, $\Psi(=V_{B,L})$ and $\Phi(=V_{B,R})$ as;

$$C_T(t)=\Theta\cdot F+\Psi\cdot\,C_{A,L}+\Phi\cdot\,C_{V,R} \tag{7}$$

Hence for fixed values of *f/p*, the resting three parameters Θ, Ψ and Φ can be estimated using standard linear least squares. The *f/p* for which the residual sum of square was minimized was determined by a direct search and associated parameter values for this solution (f, α, $V_{B,L}$, $V_{B,R}$) were obtained.

The extravascular tissue density (D_{ev} (mL/mL)) image was generated using the image reconstructed from transmission scan (*Tr*) as (Iida et al 1991):

$$D_{\mathrm{ev}} = 1.06 \cdot \left(\frac{Tr}{Tr(\mathrm{LV})} - V_{\mathrm{B}} \right) \tag{8}$$

where Tr(LV) is mean of pixel counts in LV ROI placed above, and V_B is blood volume from $C^{15}O$ scan data. PTI was generated as: PTF/D_{ev}. **(Iida et al 1991)**

References

Iida H, Rhodes CG, Silva R, Yamamoto Y, Araujo LI, Maseri A, *et al*. Myocardial tissue fraction: correction for partial volume effects and measure of tissue viability. *J Nucl Med* 1991; **32**: 2169–2175.

Iida H, Rhodes G.C, Silva R, Araujo LI., Bloomfield P, Lammertsma AA, *et al*. Use of the Left Ventricular Time-Activity Curve as a Noninvasive Input Function in Dynamic Oxygen-15-Water Positron Emission Tomography *J Nucl.Med* 1992; **33**: 1669-1677.

Choi Y, Huang SC, Hawkins RA, Kim JY, Kim BT, Hoh CK, *et al*. Quantification of myocardial blood flow using ^{13}N-ammonia and PET: comparison of tracer models. *J Nucl Med* 1999; **40**: 1045–1055.

Watabe H, Jino H, Kawachi N, Teramoto N, Hayashi T, Ohta Y, *et al*. Parametric imaging of myocardial blood flow with ^{15}O-water and PET using the basis function method. *J Nucl Med* 2005; **46**: 1219-1224.

Koeppe RA, Holden JE, Ip WR Performance comparison of parameter estimation techniques for the quantitation of local cerebral blood flow by dynamic positron computed tomography. J Cereb Blood Flow Metab. 1985; 5: 224-234.

Boellaard R, Knaapen P, Rijbroek A, Luurtsema GJ, Lammertsma AA. Evaluation of basis function and linear least squares methods for generating parametric blood flow images using ^{15}O-water and Positron Emission Tomography. Mol Imaging Biol. 2005;7:273-285.
